# Disentangling reference frames in the neural compass

**DOI:** 10.1162/imag_a_00149

**Published:** 2024-05-01

**Authors:** Léo Dutriaux, Yangwen Xu, Nicola Sartorato, Simon Lhuillier, Roberto Bottini

**Affiliations:** Center for Mind/Brain Sciences (CIMeC), University of Trento, Trento, Italy; Université Lumière Lyon 2, Laboratoire d’Études des Mécanismes Cognitifs, Lyon, France; Max Planck Institute for Human Cognitive and Brain Sciences, Leipzig, Germany; Werner-Reichardt Centre for Integrative Neuroscience, Tübingen, Germany; Graduate Training Centre of Neuroscience, IMPRS, Tübingen, Germany; LaPEA, Université Gustave Eiffel, Université Paris Cité, Versailles, France

**Keywords:** fMRI, neural compass, navigation, retrosplenial, parietal cortex, entorhinal

## Abstract

The neural system that encodes heading direction in humans can be found in themedial and superior parietal cortex and the entorhinal-retrosplenial circuit.However, it is still unclear whether heading direction in these differentregions is represented within an allocentric or egocentric coordinate system. Toinvestigate this problem, we first asked whether regions encoding (putatively)allocentric facing direction also encode (unambiguously) egocentric goaldirection. Second, we assessed whether directional coding in these regionsscaled with the preference for an allocentric perspective during everydaynavigation. Before the experiment, participants learned different object maps intwo geometrically similar rooms. In the MRI scanner, their task was to retrievethe egocentric position of a target object (e.g., Front, Left) relative to animagined facing direction (e.g., North, West). Multivariate analyses showed, aspredicted, that facing direction was encoded bilaterally in the superiorparietal lobule (SPL), the retrosplenial complex (RSC), and the left entorhinalcortex (EC), a result that could be interpreted both allocentrically andegocentrically. Crucially, we found that the same voxels in the SPL and RSC alsocoded for egocentric goal direction but not for allocentric goal direction.Moreover, when facing directions were expressed as egocentric bearings relativeto a reference vector, activities for facing direction and egocentric goaldirection were correlated, suggesting a common reference frame. Besides, onlythe left EC coded allocentric goal direction as a function of thesubject’s propensity to use allocentric strategies. Altogether, theseresults suggest that heading direction in the superior and medial parietalcortex is mediated by an egocentric code, whereas the entorhinal cortex encodesdirections according to an allocentric reference frame.

## 1 Introduction

To navigate successfully, an organism must know its current position and headingdirection. In rodents, head direction cells are thought to constitute the neuralsubstrates of facing direction. Indeed, these neurons fire in relation to theorganism’s facing direction with respect to the environment (Taube et al., 1990), working as a neural compass. Thisneural compass is suggested to be involved in the representation of goal directionduring navigation (Bicanski & Burgess,2018;Byrne et al., 2007;Erdem & Hasselmo, 2012;Schacter et al., 2012), allowing the computation of themovements required to reach a goal from the current location and orientation.

The neural compass in humans has been studied using fMRI and both univariate(adaptation) and multivariate (MVPA) approaches. These methods enabled the isolationof the brain regions representing imagined heading direction. Previous studies haverevealed a number of brain regions which represent heading direction in familiarenvironments or virtual reality. These include the entorhinal cortex, theretrosplenial complex, and superior parietal regions (Baumann & Mattingley, 2010;Chadwick et al., 2015;Chrastil et al.,2016;Kim & Maguire, 2019;Marchette et al., 2014;Shine et al., 2016,^2019^;Vass & Epstein,2013,^2017^). Whereas some of thesestudies focused on facing or goal direction in isolation (Baumann & Mattingley, 2010;Chrastil et al., 2016;Shine et al., 2016,^2019^;Vass & Epstein, 2013,^2017^), some others found that the same areas representedboth types of heading directions (Chadwick et al.,2015;Marchette et al., 2014).

Since heading directions can be aligned to environmental or absolute allocentriclandmarks (e.g., north, south), previous work has suggested that these regionsencode directions in an allocentric reference frame independent from theagent’s vantage point (Marchette et al.,2014;Shine et al., 2016;Vass & Epstein, 2017;Weisberg et al., 2018). However, in most cases,putatively allocentric heading can be accounted for by egocentric bearings tospecific landmarks (Marchette et al., 2014).For instance, when entering a new environment, it is possible to choose a principalreference vector (e.g., directed to a specific landmark or from the firstencountered perspective) and to code all directions as an egocentric bearingrelative to this vector (Shelton & McNamara,2001). Hence, since any putative allocentric direction can be expressedboth as an allocentric heading and as the egocentric angle required to rotate fromthe principal reference vector to this direction (seeFig. 1), it is still unclear whether such a directional code is encodedaccording to an egocentric or an allocentric reference frame.

**Fig. 1.**
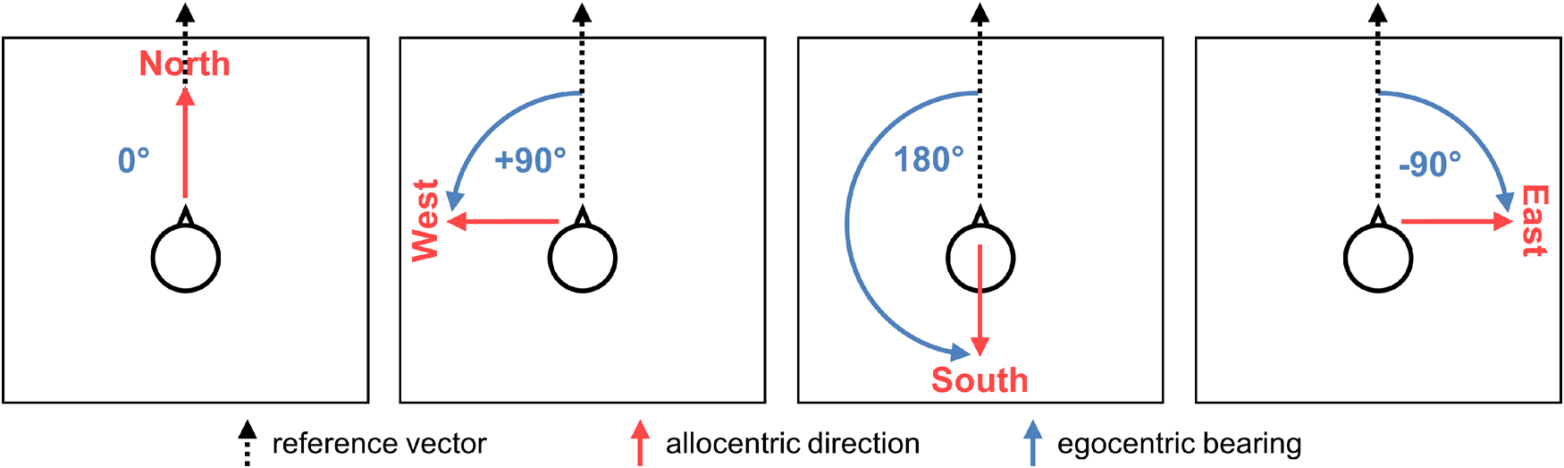
Example of how any allocentric directions (in red) can be expressed as anegocentric bearing (in blue) relative to a reference vector.

The present fMRI study aimed to disentangle these two frames of reference using fMRIand Representational Similarity Analysis (RSA) (Kriegeskorte, Mur, & Bandettini, 2008). To this end, participantswere asked to remember the location of the objects within two virtual rooms (seeFig. 2A-B). In the MRI scanner,participants were first cued to imagine themselves facing one of the four walls, andthen to recall the egocentric (goal) direction of a target object (left, right,back, front) given the current imagined facing direction.

**Fig. 2.**
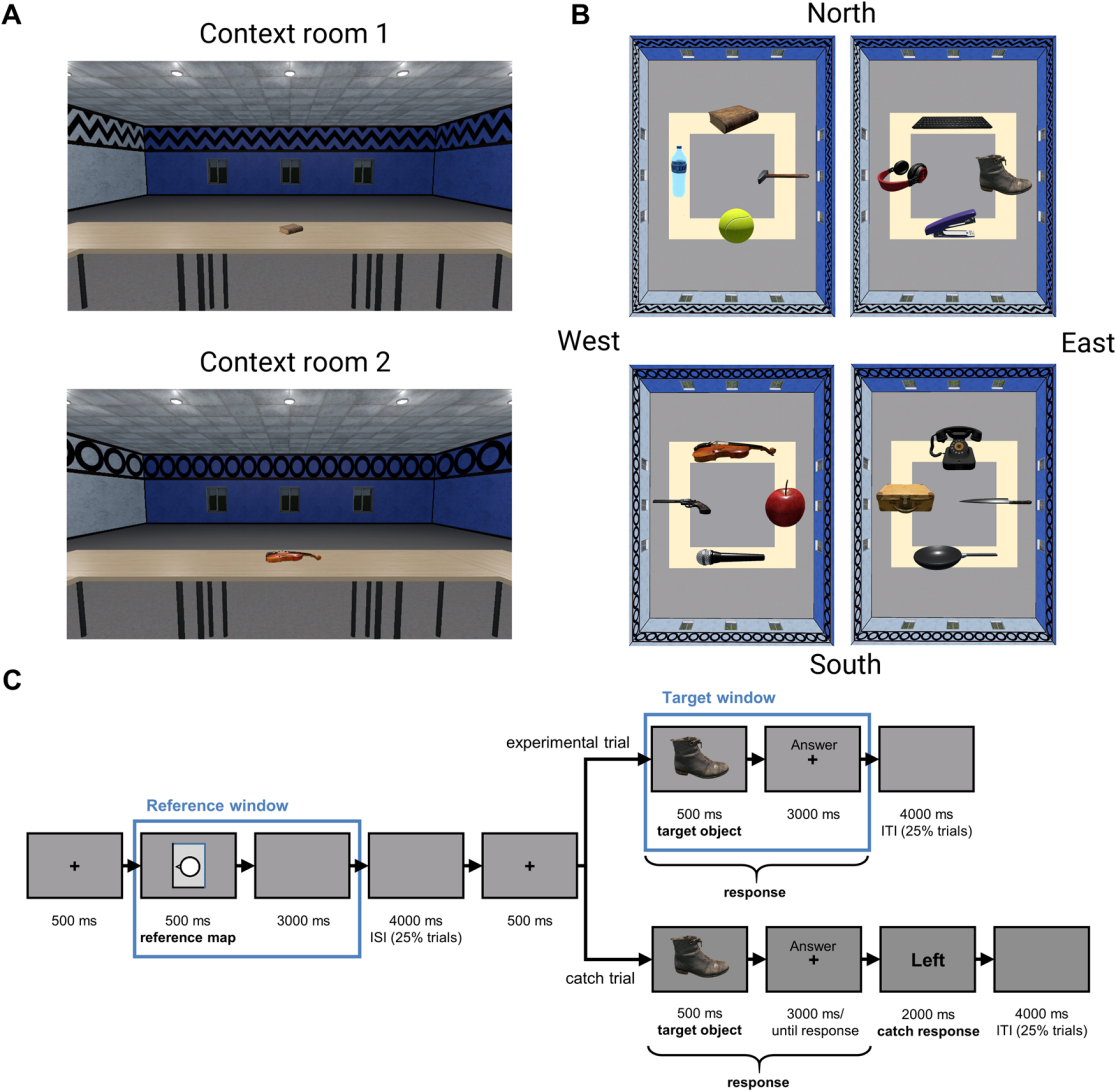
Materials. (A) Views of the two context rooms. Room 1 had a zigzag pattern onthe upper part of the walls, while Room 2 had circles. Participants were inthe middle of the room and studied it rotating their point of view clockwiseor counterclockwise using the right and the left arrow, respectively. (B)Examples of the two versions of each context room. (C) Sequence of events ofthe fMRI task. ISI, interstimulus interval; ITI, intertrial interval.

We hypothesized that heading direction is indeed encoded in the three areas ofinterest mentioned above: the entorhinal cortex (EC), retrosplenial complex (RSC),and superior parietal lobule (SPL). However, since the reference frame underlyingthe coding of heading direction is uncertain, the present study is designed todisentangle them in two different ways. First, we investigated whether the very sameregions that encoded (putatively) allocentric facing direction also encoded(non-ambiguous) egocentric goal direction. Indeed, while allocentric directions canbe expressed egocentrically with respect to a principal reference vector, anegocentric direction like left, back, or right can only be formulated with respectto the current vantage point. Second, because of the intrinsic ambiguous nature ofallocentric directions, we assessed whether these regions encode direction in anallocentric reference frame using an external validity method; namely, whether aregion was encoding allocentric heading direction as a function of thesubject’s propensity to use allocentric navigation strategies in dailylife.

## 2 Methods

### 2.1 Participants

Thirty-four right-handed native Italian speakers with no history of neurologicalor psychiatric disorders participated in this experiment (17 women and 17 men,mean age = 23.94, SD age = 3.90, range 18-35). The ethicalcommittee of the University of Trento approved the experimental protocol. Allparticipants provided informed written consent before the start of theexperiment, and they received a payment as compensation for their time. Wediscarded one participant because of a catch-trial accuracy lower than 2 SDsbelow the mean.

### 2.2 Materials

#### 2.2.1 3D rooms

Two virtual rectangular context rooms (Room 1 and Room 2) were created with*Unity*. These rooms could be distinguished based on thefrieze pattern on the upper part of the walls: Room 1 had a zigzag pattern,while Room 2 had circles (seeFig. 2A).In these rooms, one of the two short walls and one of the two long wallswere white, while the two remaining walls were blue. Hence, a wall could berecognized by its specific combination of length (short or long) and color(blue or white). The participants’ point of view was placed in themiddle of the room and was stationary. Four tables surrounded this point ofview, forming a square paralleling the room’s walls, and one objectwas placed in the middle of each of these four tables. Two differentversions of each context room were further created (Room 1 version a and b;Room 2 version a and b), containing two different object layouts todissociate object identities and object locations. In sum, in each version,the room contained a different layout with different objects. These layoutsconsisted of four objects assigned randomly to one of the four slots locatedeach in front of one of the four walls (seeFig. 2B).

#### 2.2.2 SDSR questionnaire

The SDSR (Sense of Direction and Spatial Representation) questionnaireallowed us to assess the participants’ propensity to use the surveyperspective (i.e., bird’s-eye view representations of theenvironment) during spatial navigation in everyday life (Pazzaglia et al., 2000). This questionnairecomprises 11 items on a 5-point scale (except for three items that includedonly three alternatives). Although the questionnaire provides several scoresmeasuring the propensity to use different perspectives, we were primarilyinterested in the survey perspective, which is usually associated with anallocentric frame of reference. The survey items comprised four questionsthat assessed how much participants tend to use a representation“from above” (i.e., a “map-like representation”)while navigating a city. The scores could range from -2 to 14. Although thequestionnaire addressed navigation in a large-scale environment, it has beenvalidated by its authors using a pointing task in a room (Pazzaglia et al., 2000) and has been shown to beinformative in other studies using smaller spaces (Lhuillier et al., 2018).

### 2.3 Behavioral procedures

The experiment was organized in three sessions: two training sessions—thefamiliarization and the rehearsal session—and the scanning session. Thesesessions will now be described in detail in turn. All tasks were developed withPython 3.7 using the Neuropsydia package (Makowski & Dutriaux, 2017).

#### 2.3.1 Session 1: Familiarisation session

This first training session was conducted around a week before the scanningsession. All the necessary programs and files required to conduct theexperiment were first sent to the participants, who performed it on theirpersonal laptop or desktop computer using their mouse/touchpad and keyboard.Throughout the experiment, the participants shared their screens with theexperimenter via a video call. This allowed the experimenter to giveguidance, provide instructions, and monitor the experiment’sprogress. This session was designed to familiarize participants with therooms, with the aim of constructing a mental representation of theenvironments prior to performing the main task.

Participants were presented with a room inside a virtual reality setting.They could only perform a rotation movement of their point of view from themiddle of the room by pressing the left or right arrow. No other movementinside the virtual environment was possible. When entering for the firsttime each version (a and b) of a context room (1 or 2), the vantage pointwas oriented towards the short blue wall (seeFig. 2A). For simplicity, we refer to this wall as being theNorth wall. Consistently, we associated the other walls with theircorresponding cardinal direction.

Besides studying the rooms, participants’ allocentric knowledge of therooms was assessed with three tasks (seeFig. S1). Task*a*aimed to assess participants’ knowledge of thespatial location of the objects relative to the walls of the room, whiletask*b*aimed to assess participants’ knowledge ofthe spatial location of the objects relative to the other objects. Finally,the test task was very similar to the fMRI task and was designed to ensurethat the participant would easily perform it in the scanner. The detailedsequence of tasks of this session is shown inFigure S2, and adetailed description of the training can be found in the SupplementaryMaterial.

#### 2.3.2 Session 2: Rehearsal session

The second training session was conducted in the lab, 2 days before thescanning session at the earliest. After re-studying the rooms, participantswere asked to perform the final task of the first session in the lab on ourlaptop computer. This session allowed participants to rehearse their memoryof the room before the scanning session and ensured that they could stilleasily perform the fMRI task.

#### 2.3.3 Session 3: Scanning session

Because the fMRI task was slightly different from the test task of thetraining sessions, participants were first trained to perform it beforeentering the MRI. The sequence of events of the fMRI task is shown inFigure 2C.

The timing and jitter of each trial followed the “quick”event-related fMRI design optimized for the condition-rich experiment andRSA (Kriegeskorte, Mur, & Bandettini,2008;Kriegeskorte, Mur, Ruff, etal., 2008). Each experimental trial started with a 500 msfixation cross. Then, a reference map with a character facing one of thefour walls was shown on the screen in one of the four possible orientationsfor 500 ms, followed by a 3000 ms interval. In this interval, participantswere instructed to imagine facing the wall cued by the character on thescreen. At this moment, an interstimulus interval (ISI) of 4000 ms waspresent in 25% of the trials (Zeithamova etal., 2017). Next, another fixation cross was presented for 500ms. The target object was then displayed for 500 ms, followed by a 3000 msinterval. Participants were instructed to indicate when they were ready toanswer and could do so from the beginning of the target object time windowuntil the end of the 3000 ms interval. At this moment, an intertrialinterval (ITI) of 4000 ms was present in 25% of the trials. We jittered boththe ITI and the ISI (0 or 4000 ms) to better dissociate the BOLD signalrelated to the reference and the target events (Zeithamova et al., 2017). Catch trialsrepresented 20% of total trials. These trials were identical to theexperimental trials, except that at the end, an egocentric directional word(Front, Right, Back, Left) appeared on the screen for 2000 ms. Participantshad to indicate whether this word matched the actual egocentric direction ofthe object. Fifty percent of the catch trials matched the actual directionof the object.

In the experimental trials, each object was presented in all 16 conditionsresulting from the combination of the four allocentric goal directions(i.e., the allocentric position of the target object: North, South, East, orWest) and the four egocentric goal directions (i.e., the egocentric positionof the object: Front, Back, Right, or Left) as a function of the currentfacing direction. The environment-facing direction indicated by theorientation of the character relative to the wall (allocentric—North,East, South, West) was dissociated from the screen-facing direction of thecharacter, namely, the orientation of the character relative to the screen(egocentric—top, right, bottom, and left). In other words, the mapswere presented in four different orientations across trials. The fourscreen-facing directions were counterbalanced across the 16 trials of eachof the 16 allocentric × egocentric goal direction levels. Therefore,each map orientation was presented four times for each one of these 16levels. This resulted in 256 experimental trials, to which 64 catch trialswere added. These 320 trials were arranged in 8 runs of 40 trials (32experimental trials and 8 catch trials). Each block included trials for onlyone version per context room (e.g., 1a-2a, or 1b-2a), which means that therewere two blocks for each context room × version combination (1a-2a,1a-2b, 1b-2a, and 1b-2b). Each object appeared twice in each block. Within ablock, there was a catch trial every four experimental trials on average,placed in a random position within these four trials. This was done tospread the catch trials along the whole run. The 8 catch trials within ablock were not included in the analyses and were chosen such as each of the8 objects presented in a given block was presented once, and each of the 4egocentric target positions was tested twice. Fifty percent of the catchtrials matched the actual egocentric goal direction of the object. All thiswas done to ensure that participants could not anticipate which trials werecatch trials. For the same reason, similar to the experimental trials (seeabove), 25% of catch trials comprised an ITI and 25% comprised an ISI.Participants were given the opportunity of a break between each run. Afterthe fMRI session, they had to complete the SDSR scale. To sum up, brainactivity was recorded for each participant in a within-participant design,since all participants were presented eight times for each of the 32 levelsresulting from the combination of the 2 context rooms × 4 allocentricgoal directions × 4 egocentric goal directions, and the SDSR scalewas used as a covariate four our analyses.

### 2.4 MRI procedures

#### 2.4.1 MRI data acquisition

MRI data were acquired using a MAGNETOM Prisma 3T MR scanner (Siemens) with a64-channel head-neck coil at the Centre for Mind/Brain Sciences, Universityof Trento. Functional images were acquired using the simultaneous multisliceechoplanar imaging sequence (multiband factor = 5). The angle of theplane of scanning was set to 15° towards the chest from the anteriorcommissure–posterior commissure plane to maximize the signal in theMTL. The phase encoding direction was from anterior to posterior, repetitiontime (TR) = 1000 ms, echo time (TE) = 28 ms, flip angle (FA)= 59°, field of view (FOV) = 200 mm × 200 mm,matrix size = 100 × 100, 65 axial slices, slices thickness(ST) = 2 mm, gap = 0.2 mm, and voxel size = 2 ×2 × (2 + 0.2) mm. Three-dimensional T1-weighted images wereacquired using the magnetization-prepared rapid gradient-echo sequence,sagittal plane, TR = 2140 ms, TE = 2.9 ms, inversion time= 950 ms, FA = 12°, FOV = 288 mm × 288mm, matrix size = 288 × 288, 208 continuous sagittal slices,ST = 1 mm, and voxel size = 1 × 1 × 1 mm. B0fieldmap images, including the two magnitude images associated with thefirst and second echoes of the images and the phase-difference image, werealso collected for distortion correction (TR = 768 ms, TE =4.92 and 7.38 ms).

#### 2.4.2 fMRI preprocessing

The preprocessing was conducted using the SPM12 for MATLAB ® (https://www.fil.ion.ucl.ac.uk/spm/software/spm12/). First, wecomputed each participant’s Voxel Displacement Map (VDM) using theFieldMap toolbox (Jenkinson, 2003;Jezzard & Balaban, 1995).Second, functional images in each run were realigned to the first image ofthe first run, and then the VDM were also coregistered to the first imageand were used to resample the voxel values of the images in each run tocorrect for EPI distortions caused by the inhomogeneities of the staticmagnetic field in the vicinity of the air/tissues interface. Third, thefunctional images were coregistered onto the structural image in eachindividual’s native space with six rigid-body parameters. Lastly, aminimum spatial smoothing was applied to the functional images with a fullwidth at half maximum (FWHM) of 2 mm.

#### 2.4.3 Regions of interests

Multiple ROI masks were used in the analysis. The entorhinal and the superiorparietal cortex were segmented in each subject’s native space withthe Freesurfer image analysis suite 2. The entorhinal cortex masks werethresholded at a probability of 0.5, as recommended by Freesurfer 3. Thelocation estimates for the EC were based on a cytoarchitectonic definition(Fischl et al., 2009). Given theimportance of the hippocampus in spatial cognition, we ran complementaryanalyses using a mask for the hippocampus which was also based on acytoarchitectonic definition (Iglesias etal., 2015). Since the hippocampus proper was not among thepredefined ROIs, and no significant effect was found in this region, wedecided to report these results in theSupplementaryMaterial. Masks for the SPL were based on the Destrieux atlas(Destrieux et al., 2010). Becauseactivation in RSC in previous studies was not found in the anatomicalretrosplenial cortex, we also used masks of the retrosplenial cortex definedfunctionally as category-specific regions for scene perception (Julian et al., 2012). Anatomical RSCwas defined as the combination of BA29 and 30 using MRIcron. These lastmasks were in MNI space and were then coregistered onto the structural imagein each individual’s native space.

#### 2.4.4 First-level analysis

Both the patterns instantiated during the appearance of the reference map andthe target object were analyzed. Therefore, there were 16 experimentalconditions related to the reference map (4 screen-facing directions ×4 environmental-facing directions) and 32 related to the target object (4allocentric goal directions × 4 egocentric goal directions × 2context rooms). The first-level analysis was computed using the SPM12package. The brain signal related to the reference was modeled as stickfunctions convolved with the canonical HRF, and the brain signal related tothe target was modeled as a boxcar function (with event duration equivalentto the reaction time) convolved with the canonical HRF in the time windowbetween the presentation of the target and the response. We then used theresulting T images (48 volumes, one for each condition) in the followinganalyses.

#### 2.4.5 Representational similarity analysis

##### 2.4.5.1 RDM models

Representational Similarity Analysis (RSA) uses a correlation measure tocompare a brain-based Representational Dissimilarity Matrix (RDM)obtained by calculating the pairwise correlation between patternsinstantiated in all the pairs of conditions with a model-based RDM ofexperimental interest (Kriegeskorte,Mur, & Bandettini, 2008). A brain-based RDM is asquare matrix containing the Spearman correlation distance (1−r)between two brain patterns instantiated during two different conditions.Thus, its dimension was 16 × 16 in the case of the referencewindow and 32 × 32 in the target window. The different RDMs weredesigned to detect coding for (environmental) facing direction,egocentric goal direction, and allocentric goal direction. They aredescribed in turn in the results section. Throughout the paper, whentalking about “facing direction,” we will refer to“environmental facing direction” (not screen facingdirection), unless further specified.

##### 2.4.5.2 ROI-based RSA

In the ROI-based RSA, the brain-based RDM was computed using the activitypattern of all voxels inside a given ROI. The second order-correlationsbetween the brain-based RDM of this ROI and each model-based RDM werethen performed with a partial Pearson correlation method. Although ourRDMs for facing direction and egocentric and allocentric goal directionswere only slightly correlated (all r = -.11), partialcorrelations were used to regress out confounds. For example, when aneffect was significant when computing the correlation with egocentricgoal direction, the allocentric goal direction matrix was then regressedout. Conversely, the egocentric goal direction matrix was regressed outwhen the correlations with facing directions and allocentric goaldirections were computed. In the reference window, screen facingdirection and map orientation were also regressed out from thecorrelation with the RDMs for facing directions. The resultingcorrelations were then tested to be greater than zero with a one-tailedone-sample t-test. To control for potential confounding effects of theRT, we used the duration modulation method by convolving each trial witha boxcar equal to the length of the trial’s RT for eachparticipant (Grinband et al.,2008). Moreover, partial correlation using the RTs RDM wasimplemented as a further control in some analyses.

To investigate whether individual RSA results were modulated by theparticipants’ propensity to use an egocentric or allocentricperspective, we computed the correlation between the individualsecond-order correlations for a given ROI and the individual SDSRscores. The resulting correlations were then tested to be greater thanzero with a one-tailed one-sample t-test.

##### 2.4.5.3 Searchlight-based RSA

In the Searchlight RSA analysis, a brain-based RDM was calculated foreach voxel using the pattern instantiated in the neighborhood of thevoxel of interest within a 6 mm sphere. After calculating thebrain-based RDM, we computed the second-order correlations with each RDMmodel using a partial Pearson correlation method. Similar to theROI-based RSA, egocentric RDM was regressed out for second-ordercorrelations with allocentric RDMs, and vice versa. These second-ordercorrelations were fisher z transformed to be used in the second-levelanalysis.

After computing the Searchlight images for each participant, they werenormalized using the unified segmentation method and then smoothed witha Gaussian kernel (FWHM of 6 mm) using SPM12. These normalized imageswere the input of the second-level analysis, which was performed withSnPM 13 (http://warwick.ac.uk/snpm) using the permutation-basednonparametric method (Nichols &Holmes, 2003). No variance smoothing was applied, and 10,000permutations were performed. A conventional cluster-extent-basedinference threshold was used (voxel-level at p < .001;cluster-extent FWE p < .05).

To investigate whether individual differences in allocentric strategymodulated the whole-brain activity, in the second-level general linearmodels, we used the survey score to predict the correlation between thebrain-based RDM and each model-based RDM. The resulting T-score volumefor each model-based RDM allowed us to assess where the correlation withthe model-based RDM was modulated by an individual’s propensityto use an allocentric perspective.

## 3 Results

### 3.1 Facing direction coding in the reference window is present in SPL and RSC

In the reference window, the facing direction model assumed that trials for whichparticipants had to face the same wall were similar, regardless of theroom’s orientation relative to the screen (seeFig. 3A-B). ROI analyses revealed first a significant facingdirection coding during the reference window in bilateral SPL and RSC (seeFig. 3C-F; lSPL: t(33) = 3.90, p< .001; rSPL: t(33) = 3.34, p = .001; lRSC: t(33) =1.80, p < .05; rRSC: t(33) = 2.26, p < .05), but not in EC(All ps > .05). Whole-brain analysis (whole-brain inferential statisticsare computed with primary voxel-level p < .001, cluster-level FWEcorrected p < .05) revealed an additional bilateral activation in theoccipital place area (OPA; MNI coordinate of the left peak: [-38, -80, 28],t(33) = 4.46, p_FWE_< .05; MNI coordinate of the rightpeak: [38, -76, 28], t(33) = 5.82, p_FWE_= .004; seeFig. S3). Thisresult is consistent with the hypothesis that the OPA plays an importantfunction in spatial reorientation by extracting the structure of an environmentor a scene (Julian et al., 2018).However, the exact function of OPA in navigation (perception vs memory) as wellas the frame of reference in which it operates remain unclear (See theDiscussion session for a detailed discussion of the potential role of OPA inthis study and beyond). Importantly, all these results confirm that participantshave a representation of the geometry and features of the room that isindependent of the arrays of objects in the room. No reliable correlations werefound with the SDSR scores in these analyses.

**Fig. 3.**
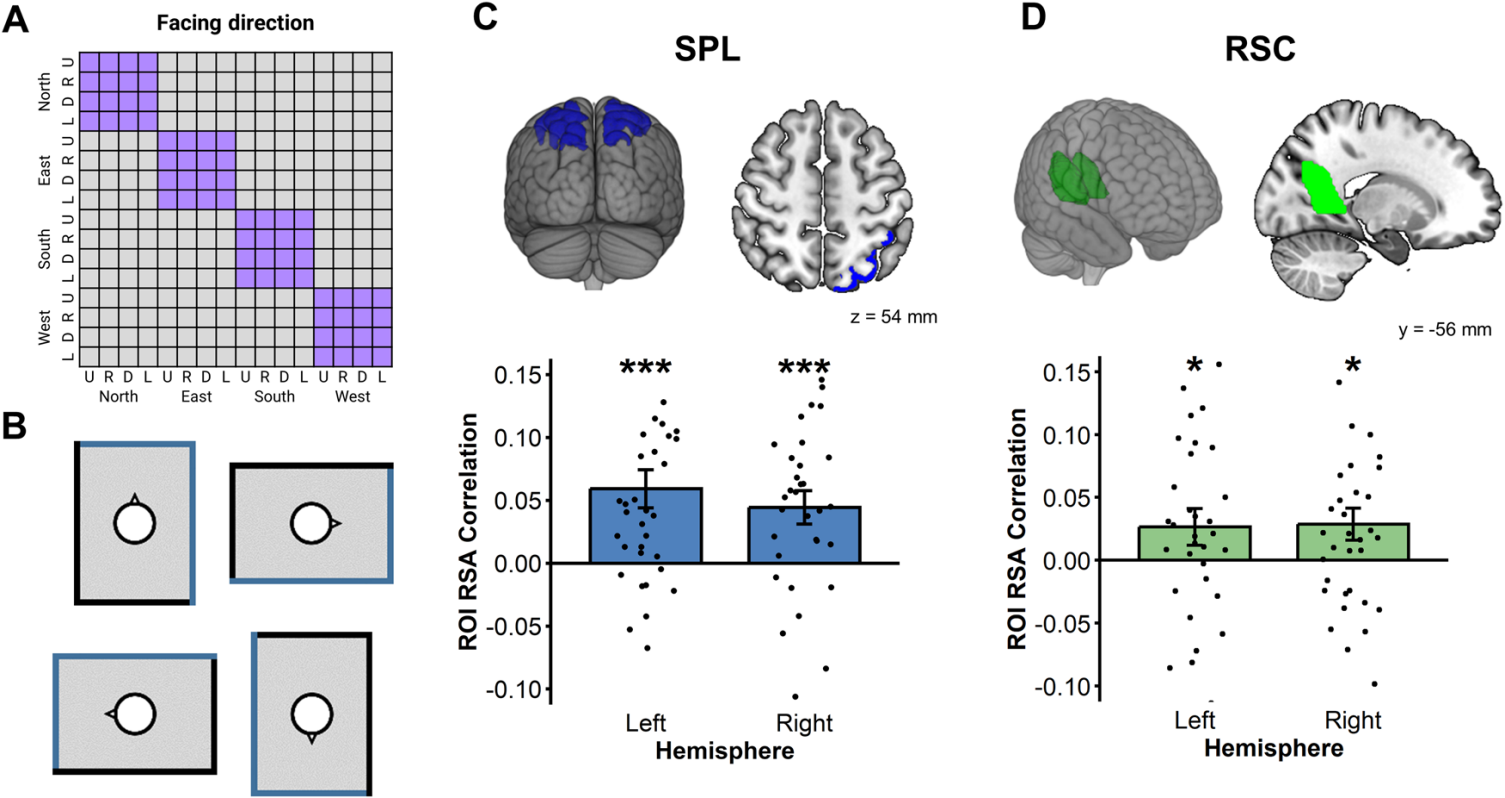
RSA for facing direction in the reference window. (A) The 16 × 16RDM for facing direction, where two trials were considered similar whenthey shared the same facing direction relative to the map, regardless ofthe orientation of the North on the screen (U = Up, R =Right, D = Down, L = Left). (B) For instance, in theseexamples, all these reference maps were cuing the same facing direction(North). (C) Both SPL showed reliable environmental facing directioncoding in the reference window. (D) Both RSC showed reliableenvironmental facing direction coding in the reference window. The errorbars represent the standard error of the mean (*p < .05,***p < .001).

### 3.2 Facing direction coding is present in the target window in the left EC

**Fig. 4.**
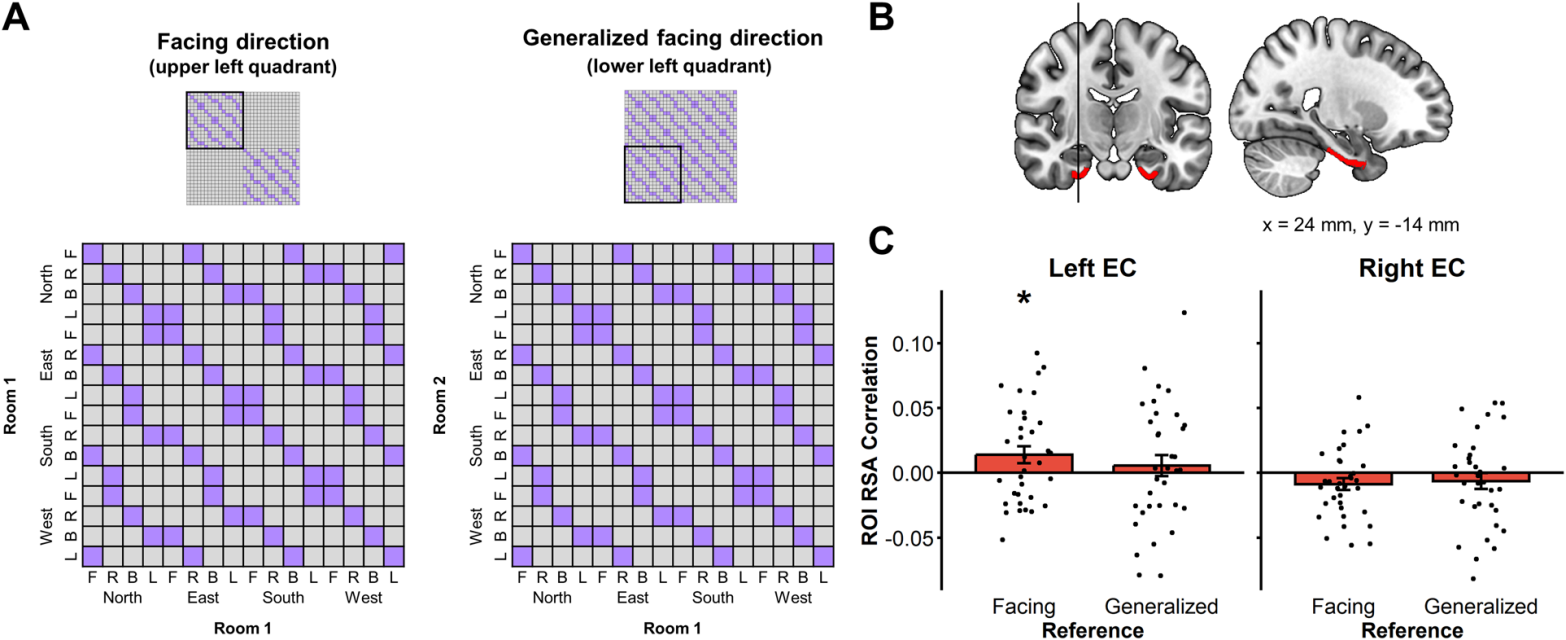
RSA for facing direction in the target window. (A) In the upper panelsare presented the whole 32 × 32 RDMs, including all Context Rooms× Allocentric goal directions × Egocentric goal directionsconditions. These matrices were indeed used on the data of the Targetwindow to detect areas that are still coding for the facing directionduring this window. For instance, it means that a trial where the targetwas North and Front was considered similar to another trial where thetarget is East and Right since it implies that the facing direction wasNorth in both cases. The black square indicates which quadrant of thematrix is represented in the lower panel. The key difference between thefacing and the facing-generalized model is that in thefacing-generalized model, two facing directions were considered similareven if they were in different context rooms (lower-right panel); whilein the facing model, they were considered similar only if they were inthe same context room (lower-left panel) (F = Front, R =Right, B = Back, L = Left). (B) EC ROIs. (C) Left ECshowed facing direction coding in the target window. For each RDM, adiamond represents the mean correlation; a box and whisker plotrepresents the median and inter-quartile range. (*p <.05).

We then investigated the encoding of facing direction in the target window. Tosolve the task, participants had to keep in memory the current facing directioncued in the reference window until the target object appeared (i.e., the targetwindow). Because the target object was presented at this moment, only then couldparticipants encode facing direction in a room- or map-specific way. Thus, forthis analysis, we created two model RDMs. In the facing direction model, onlyconditions with the same facing direction*and*the same contextroom were considered similar. In the second RDM, the facing-generalizeddirection model, facing directions were considered similar regardless of thecontext room (seeFig. 4A). A significantcorrelation with the room-specific environmental facing direction was observedin the left EC (t(33) = 2.12, p < .05; throughout the paper,p-values are corrected for multiple comparisons across the two hemispheres;Fig. 4B-C). No other ROI demonstratedroom-specific or generalized facing direction coding during the target window(All ps > .05). Whole-brain analysis did not yield any significantclusters in this case. No correlations were found with the SDSR scores in theseanalyses.

### 3.3 The parietal and retrosplenial cortex code for egocentric but not allocentric goal direction

We created three RDMs to disentangle egocentric and allocentric goal direction inthe target window (seeFig. 5). In theegocentric model, conditions in which the target object was in the sameegocentric position (e.g., to the left) were considered similar. In theallocentric model, only conditions in which the target object was placed in thesame allocentric goal direction*and*in the same context roomwere considered similar. Lastly, in the allocentric-generalized model,conditions in which the target object was placed in the same allocentric goalposition independently of the context room were considered similar. This lastRDM was designed to test whether allocentric goal direction coding generalizedacross rooms with identical geometrical layouts.

**Fig. 5.**
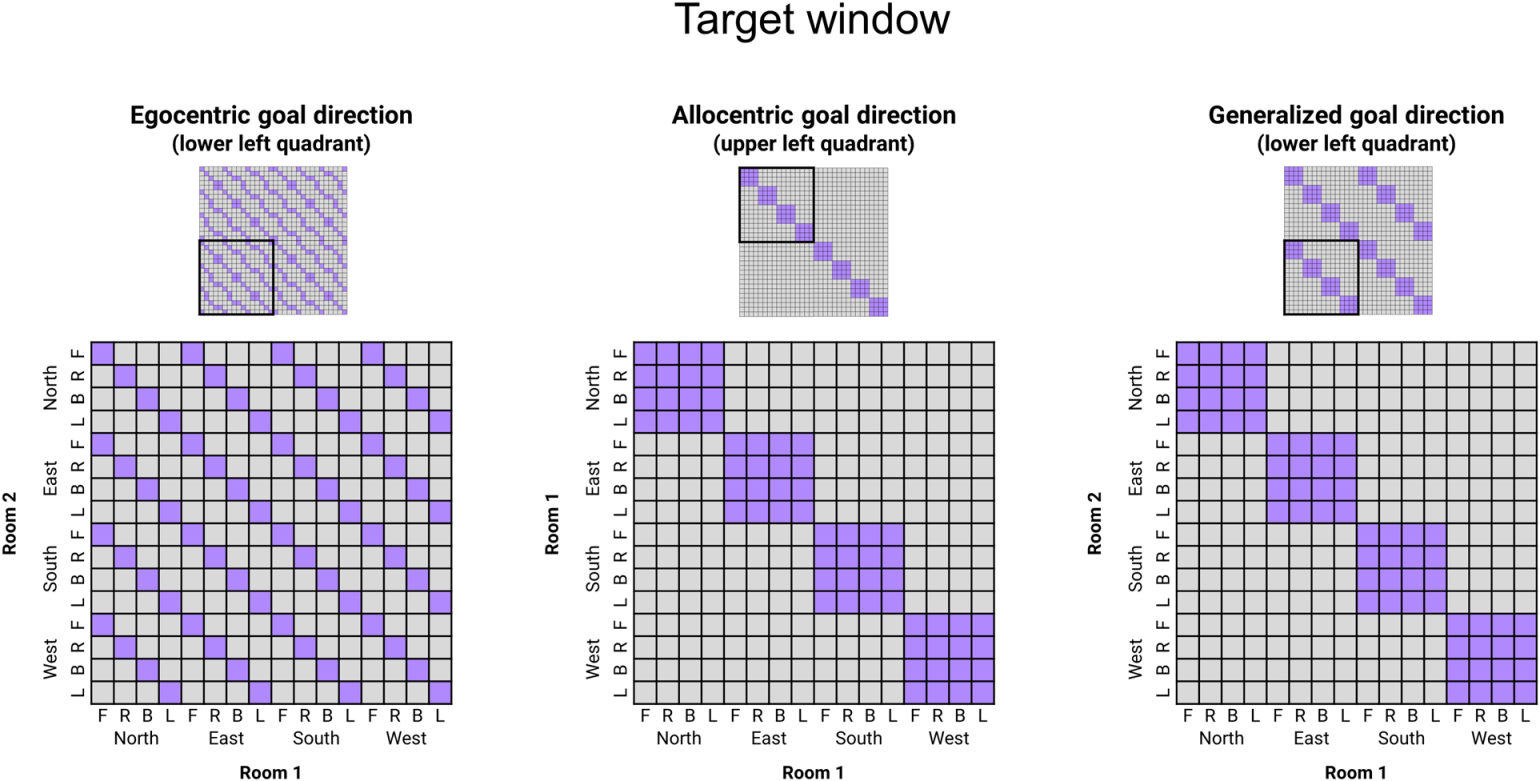
Model RDMs for target direction. In the upper panels are presented thewhole 32 × 32 RDMs, including all Context Rooms ×Allocentric goal directions × Egocentric goal directionsconditions. The black square indicates which quadrant of the matrix isrepresented in the lower panel. The key difference between theallocentric and the allocentric-generalized model is that in theallocentric-generalized model, two objects sharing the same allocentricgoal direction are considered similar even if they are in differentcontext rooms (right panel); while in the allocentric model, they areconsidered similar only if they are in the same context room (middlepanel). (F = Front, R = Right, B = Back, L =Left).

ROI analyses revealed a strong egocentric bilateral coding in both SPL and RSC(seeFig. 6A-B; lSPL: t(33) = 7.52,p < .001; rSPL: t(33) = 6.27, p < .001; lRSC: t(33)= 5.25, p < .001; rRSC: t(33) = 3.23, p = .001), butno allocentric coding (All ps > .05). No correlations were found with theSDSR scores in these ROIs, suggesting that spatial coding in the parietal cortexdid not change as a function of the propensity for a particular reference frame.Notably, this effect was also significant when we excluded the“front” condition (which, contrary to other conditions, did notrequire reorientation) and control for RTs (seeFig. S4).

**Fig. 6.**
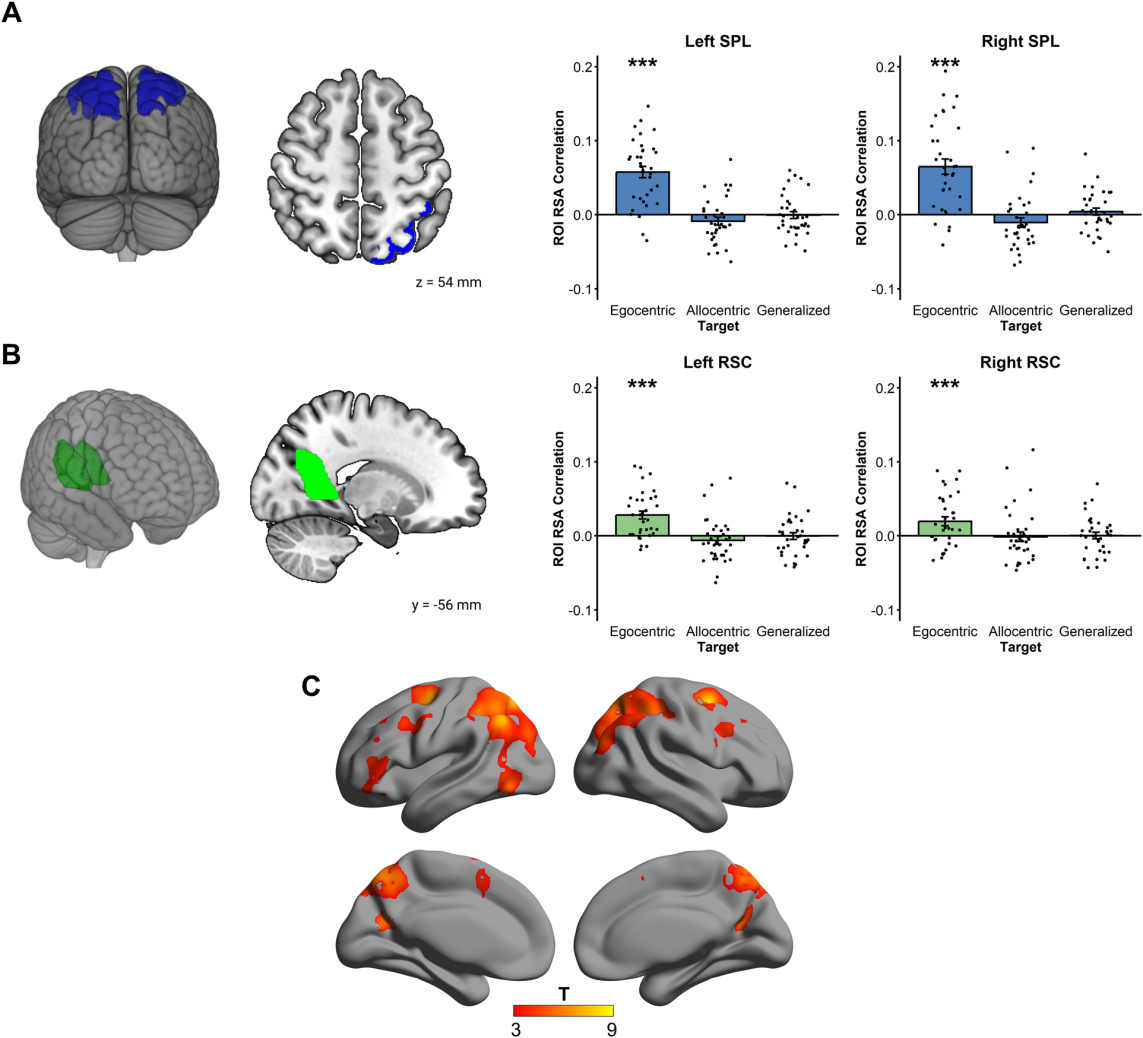
RSA for egocentric goal direction. (A) Both SPL showed reliableegocentric goal direction coding in the target window. (B) Both RSCshowed reliable egocentric goal direction coding in the target window.(C) Whole-brain searchlight RSA confirmed the large parietal involvementin representing egocentric goal direction, showing further activationsin the dorsal premotor area, the left posterior middle frontal gyrus,the left posterior cingulate cortex, and the left pars triangularis.(***p < .001; voxel level at p <.001; cluster-extent FWE p < .05).

Whole-brain searchlight RSA confirmed that the parietal cortex overall coded foregocentric goal direction (Fig. 6C),showing a very large bilateral cluster with a peak in the left AG (peak voxelMNI coordinates: [-48, -62, 44], t(33) = 8.17, p_FWE_<.001) extending in the left hemisphere to the superior parietal lobule, theprecuneus, and also ventrally in the inferior part of the occipitotemporalcortex (BA 37). It also spread in the right hemisphere to the AG, superiorparietal lobule, and precuneus. Further, two clusters were found bilaterally inthe dorsal premotor area (BA 6; left peak: t(33) = 7.56, p_FWE_= .002; right peak: t(33) = 8.98, p_FWE_= .004).Other clusters included the right and left posterior middle frontal gyrus (left:t(33) = 4.96, p_FWE_< .01; right: t(33) = 5.54,p_FWE_< .01), the left posterior cingulate cortex (t(33)= 6.80, p_FWE_< .01), and the left pars triangularis(t(33) = 4.51, p_FWE_< .01) (seeTable S1fordetails).

Next, we wanted to check whether the same voxels coding for facing direction inthe reference window also coded for egocentric goal direction in the targetwindow. For that purpose, we used the whole-brain activation maps at a lowerthreshold to extract four masks corresponding to the bilateral SPL and RSCclusters sensitive to facing direction during the reference window (seeFig. 7A-B). We then used these masks toconduct ROI analyses of the egocentric and allocentric goal direction. We foundthat the voxels coding for facing direction during the reference window in theSPL and the RSC also coded for egocentric goal direction in the target window(lSPL: t(33) = 3.46, p < .001; rSPL: t(33) = 4.87, p< .001; lRSC: t(33) = 3.08, p = .002; rRSC: t(33) =2.88, p = .004).

**Fig. 7.**
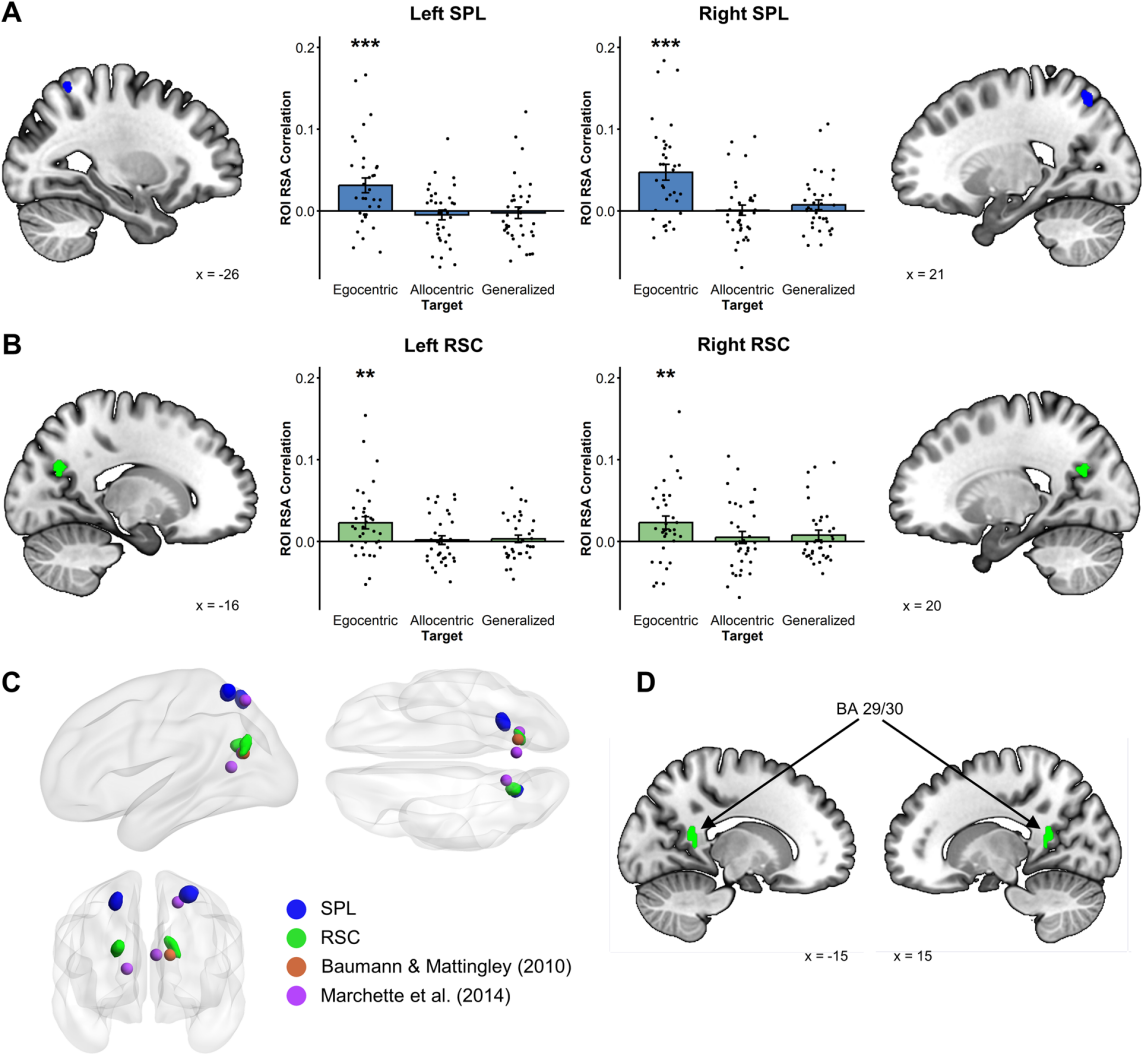
RSA Results for egocentric goal direction using functionally definedmasks where a voxel was included if it was encoding facing direction inthe reference window. (A) Voxels showing the coding of facing directionin the reference window in both SPL (extracted at p < .001)showed reliable egocentric goal direction coding in the target window.(B) Voxels showing the coding of facing direction in the referencewindow in both RSC (extracted at p < .05 for left RSC and p< .005 for right RSC) showed reliable egocentric goal directioncoding in the target window. (C) Comparisons of the location of the RSCand SPL clusters extracted from the reference window with the peakactivation coordinates inBaumann andMattingley (2010)andMarchette et al. (2014). (D) BA29/30 Masks used in thecomplementary analyses. (**p < .01,***p < .001).

We compared the exact coordinate of our brain activations with those reported inother studies observing putatively allocentric-facing direction in the superiorparietal lobule (Marchette et al., 2014)and the retrosplenial complex (Baumann &Mattingley, 2010;Marchette et al.,2014). Our activation in the SPL overlaps with the one previouslyreported byMarchette et al. (2014), andone of the masks in the retrosplenial complex overlaps with the peak of activityreported byBaumann and Mattingley (2010)(Fig. 7C). These results substantiatethe comparability of our results with previous studies reporting putativelyallocentric heading direction signals. However, our RSC masks were more lateralthan the RSC activity reported by Marchette and colleagues. Indeed, thefunctionally defined RSC used here as ROI mask (seeJulian et al., 2012and Methods section) comprises alarge portion of the medial parietal lobe, and different studies have reporteddifferent exact functional localization of the retrosplenial cortex (Baumann & Mattingley, 2010;Marchette et al., 2014;Vass & Epstein, 2017). In some studies (Baumann & Mattingley, 2010), however,heading direction coding has been reported in the anatomically defined RSC (BA29/30), which is outside the functional RSC mask used in ours and many otherstudies (Baumann & Mattingley,2010). In an exploratory analysis, we tested whether our resultsgeneralize to this region of interest. We found that facing direction in thereference window was encoded in BA 29/30 (seeFig.7Dfor representations of the ROIs) in the left hemisphere (t(33)= 2.25, p < .05; Corrected for multiple comparisons acrosshemispheres). Crucially, the same region also encoded egocentric goal directionin the target window (t(33) = 1.81, p = .04).

In sum, we performed a series of analyses using three different types of ROIs:predefined masks of the RSC and the SPL, functionally defined masks encodingfacing direction in the reference window, and anatomical masks of the RSC proper(BA 29/30). In all these cases, regions encoding putatively allocentric facingdirection in the reference window also encode unambiguously egocentric goaldirection in the target window.

### 3.4 SPL and RSC encode both facing and goal directions relative to a principal reference vector

The results presented above suggest that the SPL and the RSC code headingdirection in an egocentric fashion. One possibility is that these areas computeboth facing and goal direction through the egocentric bearing relative to aprincipal reference vector. In the case of goal direction, this reference vectorwould be the current imagined facing direction. Concerning the facing direction(reference window), it has been shown that the first experienced vantage pointin a new environment tends to be used as a reference vector from which bearingsare computed (Shelton & McNamara,2001). In the present experiment, this vantage point is in thedirection of what is called North in the present article, which is the shortblue wall (which has never been referred to as “North” to theparticipants). It is then possible that, in the SPL and RSC, both facing(reference window) and egocentric goal directions (target window), are computedegocentrically from a given reference vector. If that is the case, therepresentation of the facing direction “North” should be similarto that of the egocentric goal direction “Front.” Consequently, weshould expect the following similarity pattern between the reference and thetarget window: North = Front, South = Back, East = Right,and West = Left.

To explore this idea, we ran a new ROI-based RSA in which we computed, for eachparticipant and each ROI, the pattern similarity between the activity observedfor facing directions in the reference window and the activity observed foregocentric goal directions in the target window. This resulted in a 4 × 4matrix (seeFig. 8A) where the North, East,South, and West facing directions on one side matched the Front, Right, Back,and Left egocentric goal directions on the other side. Following the hypothesisof the principal reference vector, we expected higher average pattern similaritybetween matching directions (on the diagonal: North-Front, East-Right,South-Back, and West-Left) than between non-matching directions (off-diagonal).Because we wanted to see whether voxels coding for facing direction were codingsimilarly egocentric target direction, we used the brain masks that we extractedin the previous analyses of the reference window (Fig. 7A-B). It is important to note that results are very similarwhen the a priori anatomical/functional ROIs are used instead. Consistent withour hypothesis, average pattern similarity is higher when directions arematching than when they are not, in all parietal areas (seeFig. 8B; lSPL: t(33) = 1.86, p = .03; rSPL:t(33) = 4.69, p < .001; lRSC: t(33) = 2.43, p = .01;rRSC: t(33) = 2.13, p = .02). Importantly, we did not observe thiseffect in the left EC (t(33) = 0.32, p = .38). These resultssuggest that the same egocentric representation, anchored to a specific vantagepoint (North in the reference window and Front in the target window) is at thebasis of facing direction and egocentric goal direction encoding in the SPL andRSC. No correlations were detected with the SDSR scores in these analyses.

**Fig. 8.**
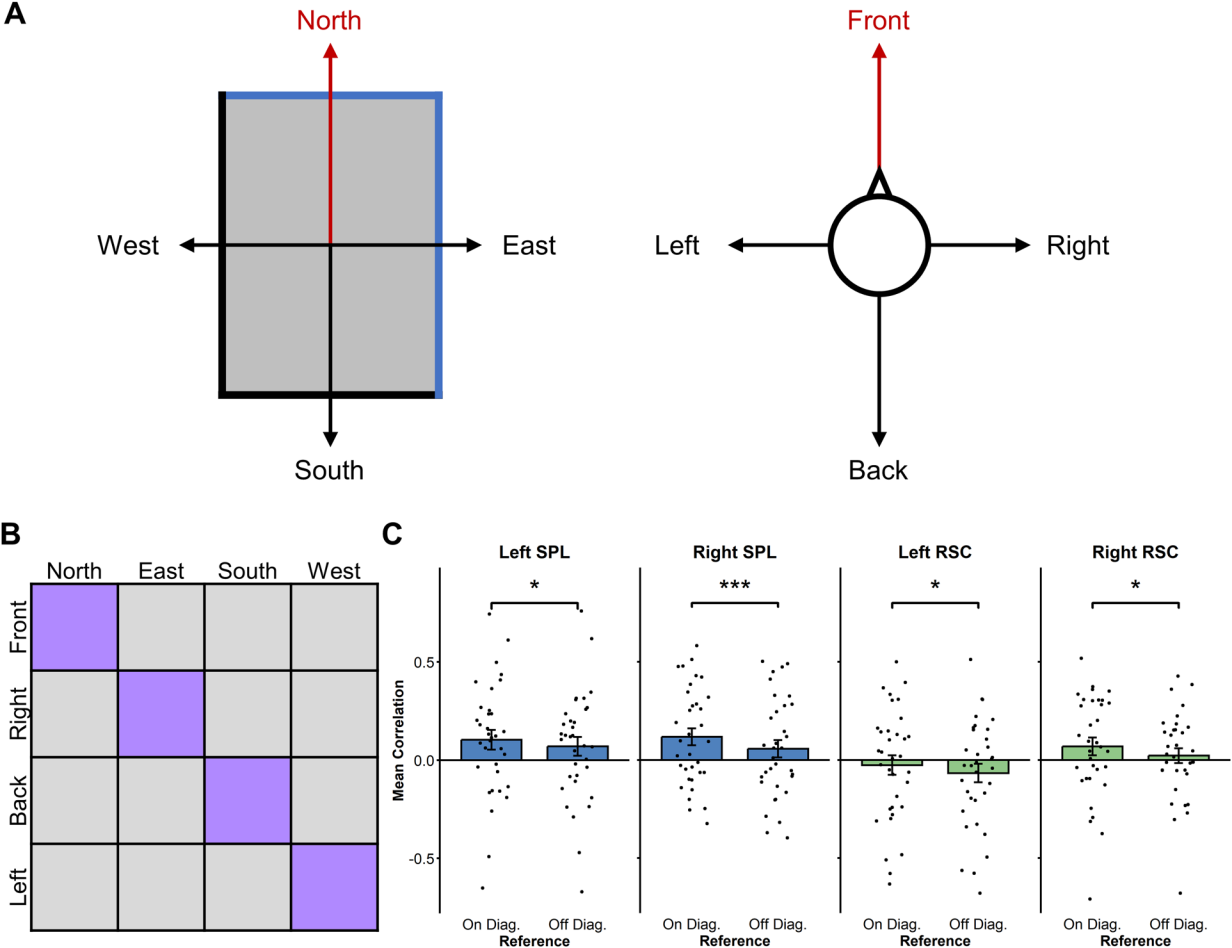
SPL and RSC both encode facing and goal directions relative to aprincipal reference vector (A) In this analysis, the reference directionNorth and the egocentric goal direction are both hypothesized asreference vectors. This means that, across reference and target windows,Front is considered as matching North, Right as matching East, Back asmatching South, and Left as matching West. (B) Brain RDMs used to testthe hypothesis that North in the reference window and Front in thetarget window are both used as principal reference vectors. In thisanalysis, we averaged the correlations of matching directions (inpurple) and the unmatching conditions (in grey) for each participant tocompare these average correlations across participants. (C) RSA resultsfor the comparison between on diagonal and off diagonal facing andegocentric target directions in SPL and RSC. Results all showed a morepositive average correlation for matching conditions (*p <.05, ***p < .001).

### 3.5 Participants’ propensity for allocentric perspective modulates goal-direction coding in the EC

The ROI analysis did not yield any reliable group-level allocentric orallocentric-generalized goal direction coding either in the EC or the parietalROIs (seeFig. S5B).On the other hand, we observed a significant modulation of the allocentric andallocentric-generalized coding in the left EC by the allocentric (survey) scoremeasured with the SDSR questionnaire (seeFig.9B-D; allocentric: r = .33, t(32) = 1.98, p <.05; allocentric-generalized: r = .38, t(32) = 2.32, p =.01). This suggests that, in our experiment, the allocentric coding in the leftEC depends on participants’ propensity to use an allocentric perspectiveduring everyday navigation.

**Fig. 9.**
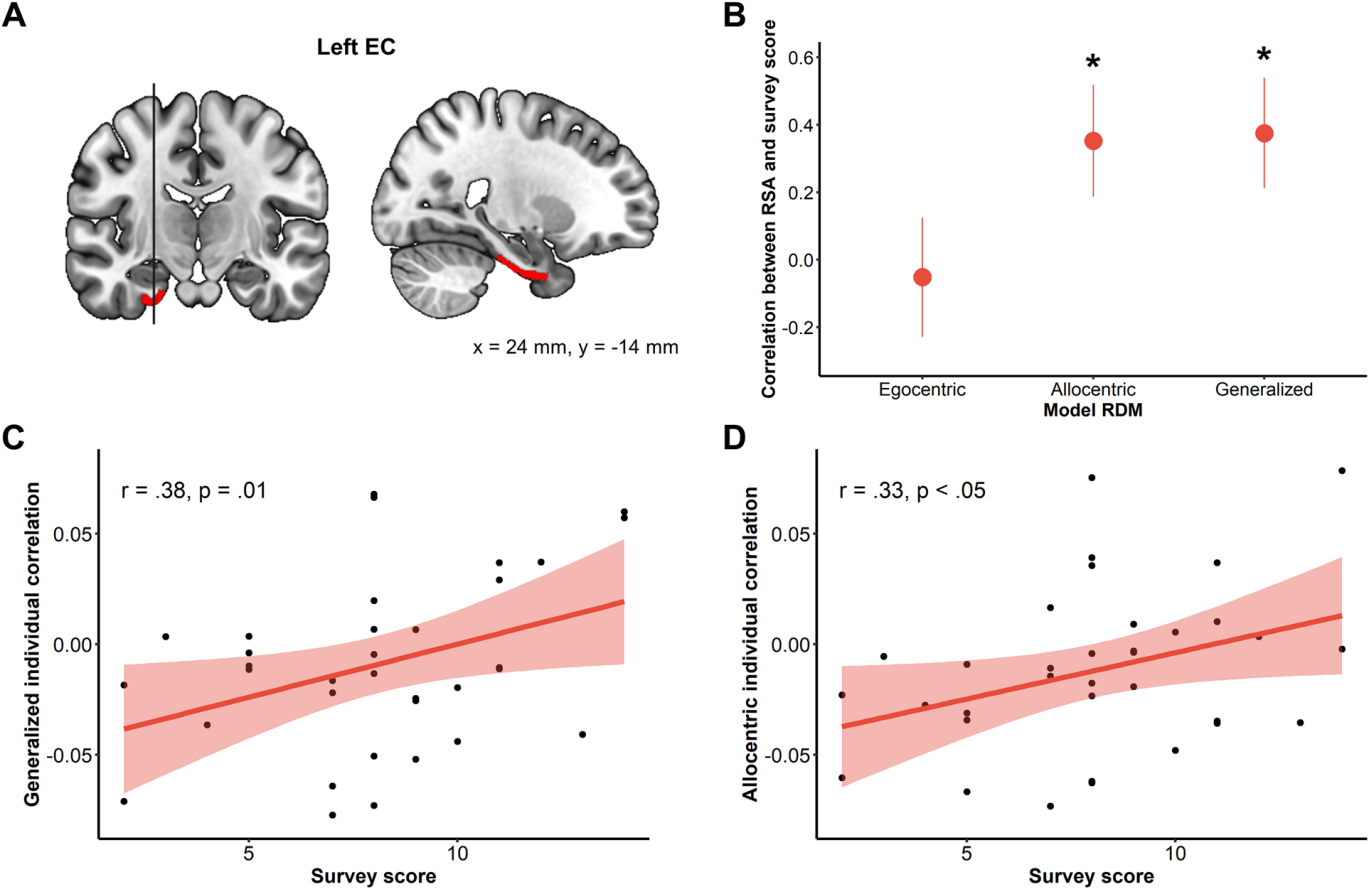
Allocentric coding in the left EC is modulated by theparticipant’s propensity for the allocentric perspective. (A)Left EC ROI. (B) Correlations between the ROI results in the left EC andthe survey score (*p < .05). (C) Scatterplot of thecorrelation between the allocentric coding in the left EC and theindividual score at the survey scale of the SDSR. (D) Scatterplot of thecorrelation between the allocentric-generalized coding in the left ECand the individual scores at the survey scale of the SDSR.

The whole-brain analysis led exclusively to bilateral occipital V1 activations(Fig. S5C), bothwith the allocentric (left: t(33) = 8.36, p_FWE_< .001;right: t(33) = 5.54, p_FWE_< .001) and theallocentric-generalized model (left: [-20, -98, 12], t(33) = 5.23,p_FWE_= .003; right: t(33) = 5.27, p_FWE_= .002) (seeTableS2for details). This was likely due either to the reactivation ofthe visual information related to the wall or to the fact that, in eachallocentric direction, the same objects are presented several times throughoutthe trials (although different objects appeared in the same allocentricdirection). Besides, contrary to the left EC, activity in V1 was not correlatedto the propensity to use an allocentric reference in everyday life (All ps> .10).

## 4 Discussion

The reference frame underlying the representation of heading direction in differentregions of the brain remains largely ambiguous. Although previous studies found thatthe entorhinal cortex (EC), the retrosplenial cortex (RSC), and the superiorparietal lobule (SPL) coded for facing and/or goal direction, they generally did notenable the disentanglement of egocentric and allocentric reference frames. Thepresent study used a reorientation task, which allowed us to address this questionby testing (i) whether the same regions that encoded (putatively) allocentric facingdirection also encoded (unambiguously) egocentric goal direction, and (ii) whetherthe activity in these regions was modulated by the subject’s propensity touse allocentric strategies in daily life. Our results confirmed first that the EC,the RSC, and the SPL all represent environmental facing direction. Up to that point,this effect could result from both allocentric and egocentric processing. However,we found that RSC and the SPL also encoded egocentric goal direction (whether anobject is on the left/right/front/back independently of its position in the map), aresult that could not emerge from an allocentric coding. Crucially, RSC and SPL didnot encode the allocentric position of the target (allocentric goal direction). Thisresult raises the possibility that these regions represent both facing and goaldirection according to an egocentric reference frame, not an allocentric one. On theother hand, the EC did not demonstrate any egocentric coding, whereas allocentricgoal direction coding in this region was uniquely modulated by participants’propensity for an allocentric perspective. Thus, in agreement with previous findings(Chadwick et al., 2015;Shine et al., 2019), the entorhinal cortex seems toencode heading direction in an allocentric reference frame. Overall, these resultssuggest that the neural compass operates in different brain regions but usingdifferent reference frames.

The present study replicated the results of previous studies finding the involvementof the EC, RSC, and SPL in facing direction coding (Baumann & Mattingley, 2010;Chadwick et al., 2015;Marchette et al.,2014;Vass & Epstein, 2013,^2017^). Our finding that the EC headingdirection system seems to operate within an allocentric reference frame is inkeeping with the hypothesis that the fMRI signal is driven, at least in part, by theactivity of head-direction cells (Taube et al.,1990). However, the fact that an egocentric reference frame provides abetter account for the heading-related activity in medial and superior parietalcortices suggests that the neural compass in these regions arises from a differentneural mechanism than the allocentric direction coded by HD cells.

One possibility is that the neural activity observed in SPL and RSC comes fromhypothetical reference vector cells, which would code for the egocentric bearingrelative to a principal reference vector (Marchetteet al., 2014). For instance, during the reference window, participantsmay take one of the walls as the principal reference vector (Shelton & McNamara, 2001) and compute the facingdirection egocentrically in reference to that wall. In the target window, thecurrent facing direction (the Front direction) could be defined as the new principalvector, and all directions would then be coded as an egocentric bearing from thisprincipal reference vector. The analysis of the similarity between the brainactivity across the reference and the target window backed this idea. Indeed, inboth SPL and RSC, we observed higher average correlations between directions thatmatched according to the reference-vector model (North = Front, East =Right, South = Back, and West = Left) than between non-matchingdirections. These findings suggest that, in the SPL and RSC, an egocentricrepresentation anchored to a specific direction (e.g., North or Front) is used toguide re-orientation for both facing direction in the reference window andegocentric goal direction in the target window. In line with these results, aprevious study that used a re-orientation task in a larger natural environment (auniversity campus) showed that putatively allocentric heading directions (North,South, East, West) were encoded in RSC both when the starting point and the targetbuildings were indicated with realistic pictures and when they were conveyedverbally. However, when the similarity between brain activity in RSC was comparedacross the two tasks (visual and verbal), only the North heading direction showed asimilar pattern across conditions (Vass &Epstein, 2017). Vass and colleagues hypothesized that the RSC preferenceto represent north-facing headings arose because the RSC represents environmentsaccording to a particular reference direction (McNamara et al., 2003;Mou et al.,2004;Waller & Hodgson,2006). Although such direction was suggested to be computedallocentrically, Vass and colleagues could not establish which frame of referencewas actually utilized. Here, we observed that the reference vector is updateddepending on the imagined position of the body. Thus, this study not only supportsthat, in the RSC and SPL, heading is derived relative to a reference vector, butalso that this computation is done within an egocentric frame of reference.

In this paper, we also showed that the representation of allocentric goal directionin the left EC was modulated by participants’ propensity for the allocentricperspective in everyday life. This, together with the presence of facing directioncoding and the absence of egocentric coding, suggests that the EC coded for headingdirection in an allocentric frame of reference (see alsoChadwick et al., 2015). Consistently, the entorhinalcortex has strongly been associated with allocentric representation in theliterature, particularly through the presence of grid cells (Hafting et al., 2005), which are thought to provide thescaffolding of allocentric representations (Buzsáki & Moser, 2013). Contrary to previous results(Chadwick et al., 2015;Shine et al., 2019), we did not find a consistentrepresentation of allocentric goal direction in the entorhinal cortex acrosssubjects (i.e., independently from their everyday navigation style). One possiblereason for this discrepancy is that we did not explicitly ask subjects to providethe allocentric location of the target object (North, South, East, West) during thetask, but only the egocentric one (Front, Back, Right, Left). Thus, participantscould solve the task relying solely on egocentric information. Our result suggeststhat the activation of an allocentric map to retrieve the position of objects is notautomatic. This interpretation is in line with previous studies showing thatdifferent cognitive styles in spatial strategies lead to the activation of partiallydifferent neural networks during the same spatial task (Iaria et al., 2003;Jordan et al., 2004). We might have failed to observe allocentric goaldirection coding in the RSC for similar reasons. Indeed, according to a prominentspatial memory model (Bicanski & Burgess,2018;Byrne et al., 2007), the RSCshould serve as a hub where spatial information is transformed across referenceframes. If that is the case, one should expect to find both allocentric andegocentric goal direction coding in this region. Nevertheless, if the activation ofan allocentric map is indeed not necessary for the task, reference framestransformation might not have been necessary either.

The absence of locomotion might have had an impact on our capacity to detect genuineHD cell signals. For instance, in the paradigm used byChadwick et al. (2015), participants were required toface various allocentric directions from different viewpoints. Additionally, theconstraints on head movement within the MRI setting might have obstructed ourability to detect HD cell activity, given that the vestibular input was notinformative during the task. However, it is worth noting that the HD signal has beenobserved to update based on visual landmarks even when head movement is restricted,as demonstrated by previous research (Jeffery etal., 2016;Yoder et al., 2011).This suggests that the present experimental design should have theoretically enabledus to identify typical HD signals.

The environments used in this experiment did not allow us to disentangle between thecoding of allocentric direction and environmental boundaries, due to the fact thateach allocentric direction was associated with a specific wall (which is notuncommon in fMRI studies on heading direction; e.g.,Chadwick et al., 2015;Shine et al.,2019). This could explain why facing direction in the reference windowwas also encoded in the Occipital Place Area (OPA), which is involved inrepresenting environmental boundaries during visually guided navigation (Julian et al., 2016). However, additionalunplanned analyses have shown that the OPA also encodes egocentric goal directionduring the target window (Fig.S6), but not allocentric goal direction (which would be akin to encodingthe position of an object tethered to a specific boundary). Moreover, RSA analysisacross the two temporal windows showed a similar mapping of facing direction ontoegocentric goal direction as showed for RSC and SPL (Fig. S6). Therepresentation of egocentric environmental structure in this brain region isconsistent with previous fMRI studies in which the OPA showed different levels ofactivation for a picture of a scene and its mirror image (left vs right sensitivity;Dilks et al., 2011) and encodedegocentric distance (Persichetti & Dilks,2016) and egocentric perspective motion (Kamps et al., 2016) implied by visual scenes. The role of OPA beyondvisually guided navigation as well as the reference frame in which it operates,however, remains unclear. Although our study was not designed to address thisspecific question, the fact that, in our paradigm, OPA encodes memory retrievedegocentric goal direction (but not boundary-tethered allocentric goal direction),and encodes both goal and facing direction relative to an egocentric referencevector, suggest the involvement of OPA in spatial reorientation from memory,operating in an egocentric reference frame.

Finally, it is important to note that in the present paradigm, participants werestudying the environment from a single vantage point, without any locomotion. Suchspaces are often referred to as vista spaces, as opposed to environmental spaceswhere locomotion is necessary to explore the environment (Montello, 1993). Previous research on the neural compassmade use of both vista spaces (Chadwick et al.,2015;Shine et al., 2016) andenvironmental spaces (Baumann & Mattingley,2010;Kim & Maguire, 2019;Marchette et al., 2014;Shine et al., 2019;Vass& Epstein, 2013,^2017^).However, there are both qualitative and quantitative differences in the vista orenvironmental space. For instance, spatial memory in vista space is more sensitiveto the intrinsic properties of the layout (Meilingeret al., 2016) and leads to better pointing performance (He et al., 2019). Further, while allocentric-relatedactivity in the EC has been observed in both environmental (Shine et al., 2019) and vista space (Chadwick et al., 2015), it has been shown that thepresence of visual barriers in a room modulate grid-like signal in the EC (from a6-fold to a 4-fold symmetry) (He & Brown,2019). A similar argument can be made for large and small environments,that also can induce different navigational strategies (Burgess, 2006;Hegarty etal., 2006; but seeLawton, 1996).Altogether, these studies suggest that extrapolating our conclusions fromobservations in a small vista space to other types of environments should be madewith caution, although there is evidence that principal reference vectors are usedin both vista and environmental spaces (Meilinger etal., 2014).

## 5 Conclusion

Overall, the present work allowed us to disentangle between different referenceframes supporting the representation of heading direction across different brainregions. We showed that superior and medial parietal regions encode not only facingdirection, as already suggested in previous studies (Baumann & Mattingley, 2010;Marchette et al., 2014;Vass &Epstein, 2017), but also egocentric goal direction. This finding suggeststhe use of a common egocentric reference frame to represent heading direction inthese regions. On the other hand, no egocentric coding emerged in the entorhinalcortex, which, beyond representing facing direction, also represents allocentricgoal direction as a function of the individual propensity to use allocentricnavigational strategies in everyday life. Although limited to a particular spatialsetting (small environments without translation or actual head rotation of theobserver;Shine et al., 2016), our studyhighlights the necessity to investigate how different brain regions may encodesimilar spatial features by means of different computations across reference frames.Beyond space, one might ask whether the same sort of mechanism would apply innon-spatial domains. Indeed, recent works have suggested that the EC and the PC canbe used to “navigate” non-spatial domains, particularly conceptualdomains (Bellmund et al., 2018), acrosscomplementary reference frames (Bottini &Doeller, 2020;Viganò et al.,2023).

## Supplementary Material

Supplementary Material

## Data Availability

Our code is publicly available athttps://github.com/BottiniLab/allo-ego, and data are available from thecorresponding author upon request, without restriction.
