# Supplementary material for: Disentangling reference frames in the neural compass
Source: Imaging Neurosci (Camb). 2024 May 1;2:imag-2-00149. doi: 10.1162/imag_a_00149 (PMC12247608; doi:10.1162/imag_a_00149)
Supplement: Supplementary Material [file imag_a_00149-supp.pdf]

Detailed training procedure

The experiment was organised in three sessions: two training sessions – the familiarisation and the rehearsal session – and the scanning session. These sessions will now be described in detail in turn. All tasks were developed with Python 3.7 using the Neuropsydia package (Makowski and Dutriaux, 2017).

1.1.1. Session 1: Familiarisation session

This first training session was conducted around a week before the scanning session. All the necessary programs and files required to conduct the experiment were first sent to the participants, who performed it on their personal laptop or desk computer using their mouse/touchpad and keyboard. Throughout the experiment, the participants shared their screens with the experimenter via a video call. This allowed the experimenter to give guidance, provide instructions, and monitor the experiment's progress effectively. This session was designed to familiarize participants with the rooms to allow them to construct a mental representation of the environments and to train them to perform the main task. Besides studying the rooms, participants’ allocentric knowledge of the rooms was assessed with three tasks (see Figure S1). Task *a* aimed to assess participants’ knowledge of the spatial location of the objects relative to the walls of the room, while task *b* aimed to assess participants’ knowledge of the spatial location of the objects relative to the other objects. Finally, the test task was designed to ensure that the participant would easily perform the fMRI task.

The detailed sequence of tasks of this session is shown in Figure S2. Participants were first asked to study both versions of one room and to perform task *a* to test their memory of this room. They had then to repeat the same procedure with the other room. Second, the participants had to perform the same sequence of study phase and memory test with task *b*. Third, after

participants were given the opportunity to study all four versions another time, they had to perform a version of tasks *a* and *b* that tested their memory of all rooms at once. The order of study of each room and the order of study of versions a and b of each room were counterbalanced across participants. This resulted in four possible study orders. Importantly, if the participant's accuracy was lower than 85% at one of the tasks, they were asked to study again the room versions related to this task and to perform it again. If needed, they had to repeat the study/testing sequence until they reached the threshold of 85%. The study phase and the three tasks will now be detailed in turn.

1.1.1.1. Study phase

To familiarize the participants with the virtual environment set up, they were instructed to study an empty room with no pattern on the wall as an example. To study a room, they could only perform a rotation movement of their point of view from the middle of the room by pressing the left or right arrow. No other movement inside the virtual environment was possible. The first study of each version of the rooms lasted two minutes, and participants were asked to memorize the objects and their position. Any later study of the rooms lasted a maximum of one minute. When entering for the first time a version of a room, the vantage point was oriented towards the short blue wall (see Figure 2A). For simplicity, we refer to this wall as being the North wall. Consistently, we associated the other walls with their corresponding cardinal direction.

1.1.1.2. Task a

As represented in Figure S1A, a trial of this task consisted of the presentation of a schematic map of a room on the left and of a target object on the right. The map was rectangular and included all information about the walls, but no information about the objects, as four white squares were placed on the map at all four possible object locations. Using the mouse,

participants had to indicate with a left click the white square corresponding to the object's location relative to the walls. Task *a* comprised 16 trials after studying a single room, and 32 after studying both rooms. Each object was presented as target twice in random order. The map was presented in 4 different orientations (i.e., the north oriented towards the top, the right, the bottom, or the left of the screen), and this orientation was counterbalanced across trials.

1.1.1.3. Task b

Similar to task a, a trial of this task consisted of the presentation of a schematic map of a room on the left and of a target object on the right. Different from task *a* however, the walls were not displayed, and one reference object was placed at one of the four possible locations (see Figure S1B). Participants had to indicate with the mouse the target object's location relative to the reference object. Task *b* comprised 16 trials after the study of a single room, and 32 after the study of both rooms. Each object in the room was a target twice, and objects were presented in random order. The reference object was chosen randomly, and the orientation of the map on the screen was counterbalanced.

1.1.1.4. Test task

An example of a trial is presented in Figure S1C. A trial started with the presentation of a fixation cross for 2000 ms. Then, a reference map with a character facing one of the four walls was shown on the screen in one of the four possible orientations for 500 ms. At this time, participants were instructed to imagine facing the wall cued by the character on the screen. Immediately after the map, a fixation cross was displayed along with the word "Ready". Participants had to press the space bar when they finished imagining which triggered the disappearance of the word. After 3500 ms from the outset of the map, the target object was displayed for 500 ms, followed by a 3500 ms screen prompting them to answer. They then had a

total of 4000 ms to indicate the egocentric position of the object relative to their imagined heading (i.e., front, back, left, or right) using the directional arrows on the keyboard. During this task, each object was presented eight times, twice for each of the four egocentric directions. This resulted in 128 trials, which were presented randomly in 4 blocks of 32 trials. The four orientations of the reference map were counterbalanced across the eight trials of each of the 16 allocentric × egocentric levels. Therefore, each map orientation was presented twice for each of these 16 levels. In addition, each object appeared twice in a block. There was a one-minute break between each block. Only participants that scored at least 85% on this test were considered for the subsequent phases.

Results for the ROI-based RSA in the hippocampus

We first analyzed facing direction coding in the hippocampus (HPC) using RSA in the reference window. The facing direction model assumed that trials for which participants had to face the same wall were similar, regardless of the room's orientation relative to the screen (see Figure 3A-B). ROI analyses did not reveal any significant facing direction coding in either hippocampus (lHPC: $t(33) = -0.03$, $p = .51$; rHPC: $t(33) = -0.78$, $p = .78$).

Next, we analyzed facing direction during the target window. In the facing direction model, only conditions with the same facing direction *and* the same context room were considered similar; while in the facing-generalized direction model, facing directions were considered similar regardless of the context room (see Figure 4A). Results did not show any correlations between HPC activity with either the facing direction RDM (lHPC: $t(33) = -0.15$, $p = .56$; rHPC: $t(33) = 1.64$, $p = .06$) or the facing-generalized direction RDM (lHPC: $t(33) = 0.57$, $p = .29$; rHPC: $t(33) = 0.95$, $p = .17$).

Finally, we tested for egocentric, allocentric, and egocentric and allocentric-generalized goal directions in the target window (see Figure 5). In the egocentric model, conditions in which the target object was in the same egocentric position (e.g., to the left) were considered similar. In the allocentric model, only conditions in which the target object was placed in the same allocentric goal direction *and* in the same context room were considered similar. In the allocentric-generalized model, conditions in which the target object was placed in the same allocentric goal position independently of the context room were considered similar. No correlation was observed between the activity in the HPC and the egocentric model (lHPC: $t(33) = -0.24$, $p = .60$; rHPC: $t(33) = 0.11$, $p = .46$), allocentric model (lHPC: $t(33) = -1.45$, $p = .92$; rHPC: $t(33) = 0.34$, $p = .63$), or allocentric-generalized model (lHPC: $t(33) = -1.69$, $p = .95$; rHPC: $t(33) = -1.69$, $p = .95$).

For each of these three analyses, we also assessed whether the observed correlations between hippocampal activity and our RDMs were modulated by the allocentric (survey) score as measured with the SDSR questionnaire. No significant modulation was found in any of these analyses (All $ps > .08$).

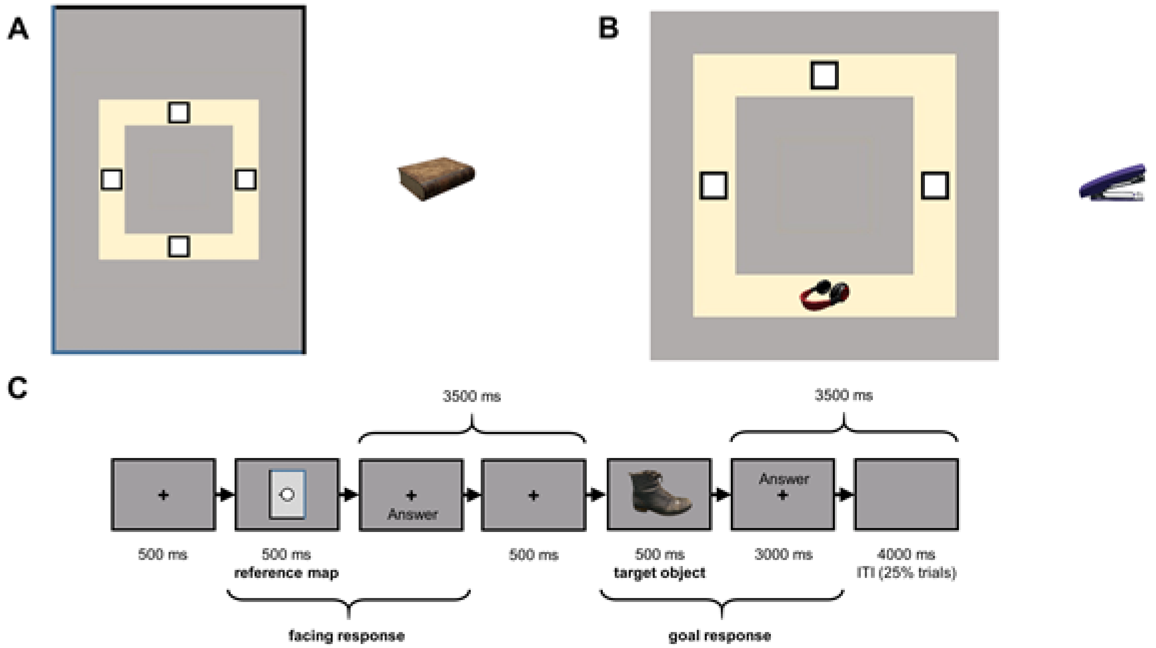

Figure S1. *Tasks of the training sessions. (A) Task a required participant to click on the right location considering the orientation of the room indicated by the walls. (B) Task b required participants to click on the right location considering the position of a reference object already placed on the table. (C) The sequence of events of the test phase in sessions 2 and 3 that were designed to train participants to perform the fMRI task.*

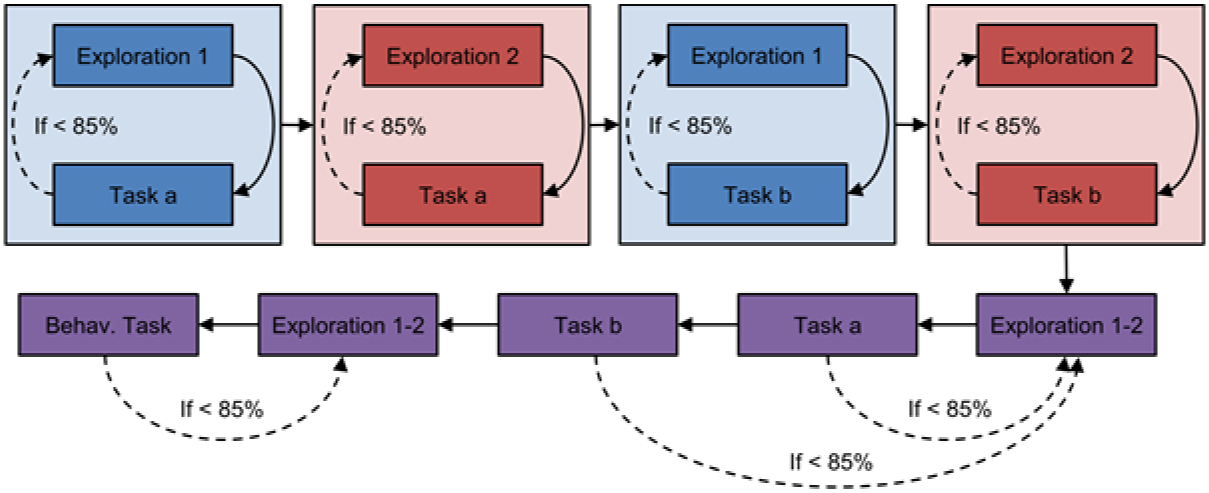

Figure S2. *Flowchart depicting the structure of the online training session. Exploration 1 refers to the exploration of the two versions of one of the rooms, exploration 2 refers then to the exploration of the two versions of the other room, and Exploration 1-2 refers to the exploration of both versions of both rooms. For each task, if participants were not able to have an 85% accuracy, participants had to perform again the exploration.*

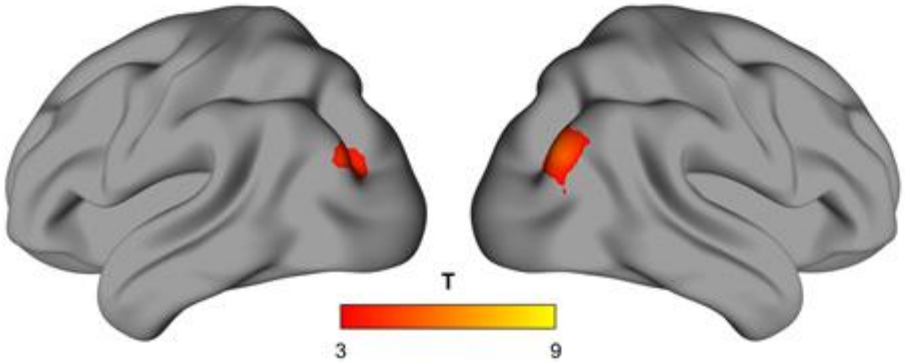

Figure S3. *Whole-brain searchlight RSA during the reference window showed a bilateral activation of the occipital place area related to facing direction. A conventional cluster-extent-based inference threshold was used (voxel level at* $p < 0.001$*; cluster-extent FWE* $p < 0.05$*).*

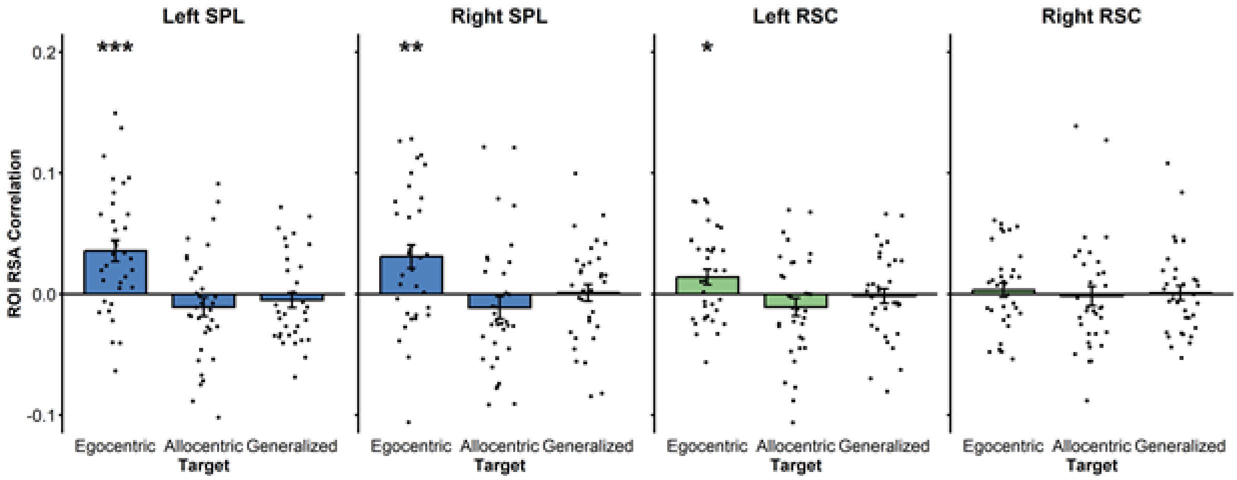

Figure S4. *ROI analyses of the egocentric target direction in the parietal ROIs excluding the Front direction and controlling for response times (* p < .05, ** p < .01, *** p < .001).*

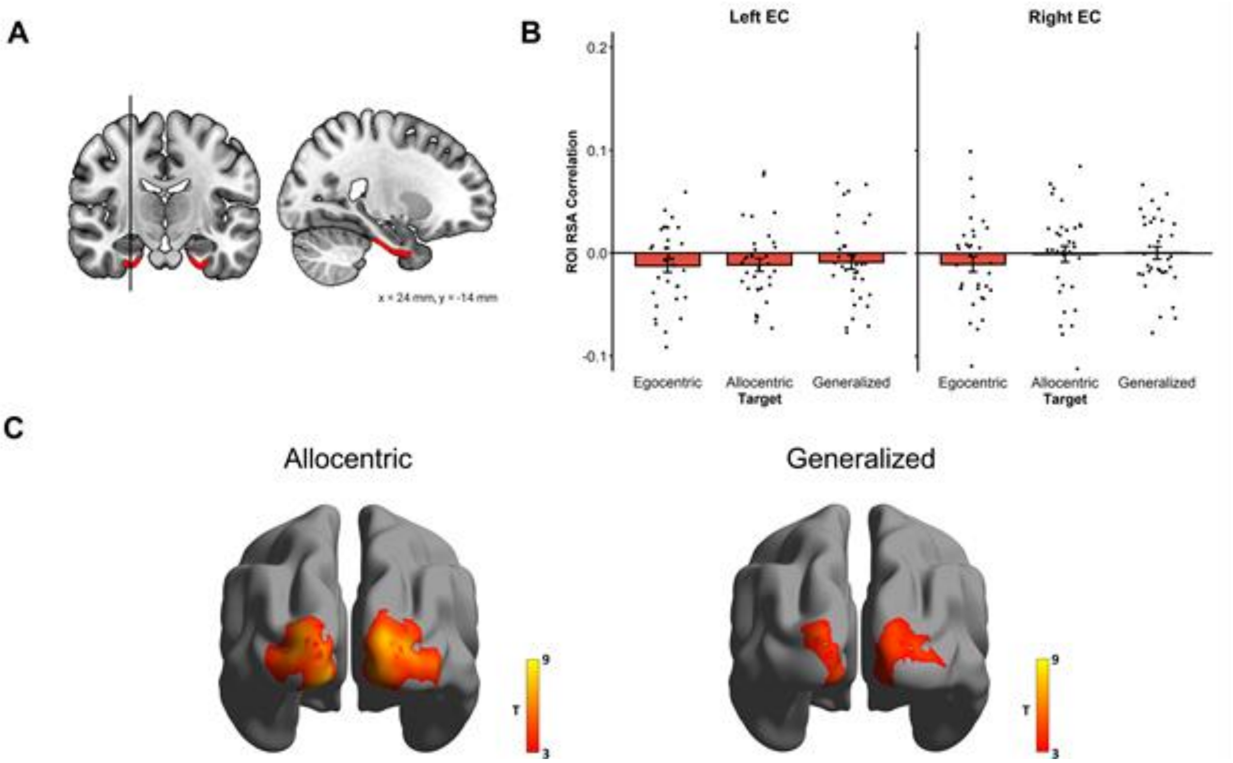

Figure S5. *RSA Results for the allocentric goal direction. (A) EC ROIs (B) No reliable allocentric nor egocentric coding was observed in the ECs. (C) Allocentric and Allocentric-generalized whole-brain searchlight RSA results.*

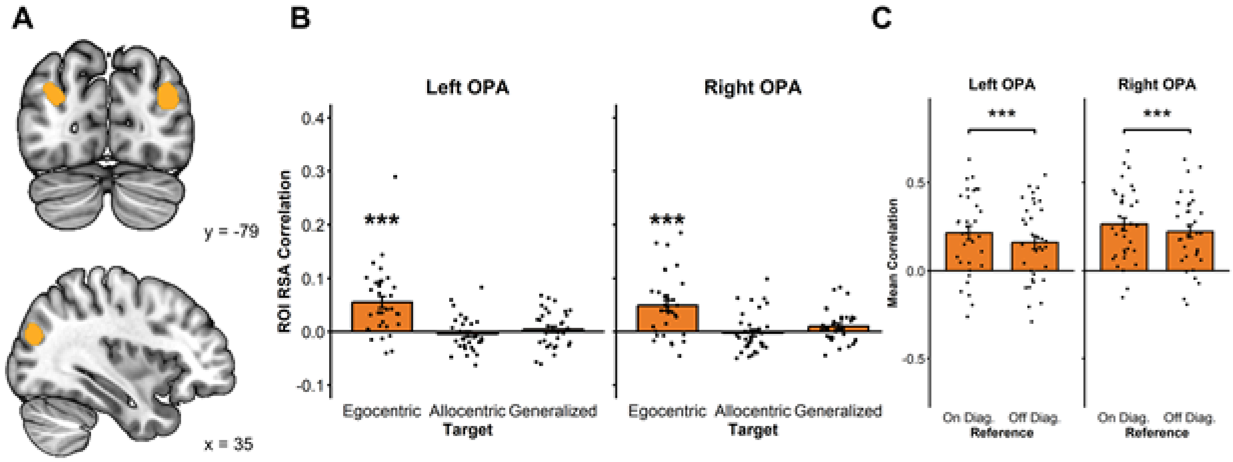

Figure S6. *Exploratory RSA Results for OPA. (A) OPA ROIs (B) Both OPA showed reliable egocentric goal direction coding in the target window. (C) RSA results for the comparison between on diagonal and of diagonal reference and egocentric target directions in OPA. Results all showed a more positive average correlation for matching conditions (*** p < .001).*

Region	MNI coordinates			Cluster size (mm^3)	t	p_{FWE}
	x	y	z			
L angular gyrus	-48	-62	44	103680	8.17	< .001
L dorsal premotor area	-26	-8	58	11776	7.56	.002
L posterior middle frontal gyrus	-46	4	36	5112	4.96	.008
L posterior cingulate cortex	-16	-62	20	3136	6.80	.02
L pars triangularis	-48	40	8	6344	4.51	.006
R dorsal premotor area	24	0	54	8192	8.98	.004
R posterior middle frontal gyrus	46	18	32	5544	5.54	.002

Table S1. *Results of the egocentric whole-brain analyses. A conventional cluster-extent-based inference threshold was used (voxel level at $p < 0.001$; cluster-extent FWE $p < 0.05$).*

Region	MNI coordinates			Cluster size (mm^3)	t	p_{FWE}
	x	y	z			
**Allocentric**						
L occipital cortex	-16	-88	-12	17088	8.36	< .001
R occipital cortex	18	-98	10	19928	5.54	< .001
**Generalized**						
L occipital cortex	-20	-98	12	6128	5.23	.003
R occipital cortex	16	-96	12	9800	5.27	.002

Table S2. *Results of the allocentric and allocentric generalized goal whole-brain analyses. A conventional cluster-extent-based inference threshold was used (voxel level at $p < 0.001$; cluster-extent FWE $p < 0.05$).*
